# The multi-faceted roles of R2TP complex span across regulation of gene expression, translation, and protein functional assembly

**DOI:** 10.1007/s12551-023-01127-9

**Published:** 2023-09-12

**Authors:** Sifiso Duncan Luthuli, Addmore Shonhai

**Affiliations:** Department of Biochemistry and Microbiology, University of Venda, Thohoyandou, South Africa

**Keywords:** R2TP complex, RUVBL1, RUVBL2, Protein complex

## Abstract

Macromolecular complexes play essential roles in various cellular processes. The assembly of macromolecular assemblies within the cell must overcome barriers imposed by a crowded cellular environment which is characterized by an estimated concentration of biological macromolecules amounting to 100–450 g/L that take up approximately 5–40% of the cytoplasmic volume. The formation of the macromolecular assemblies is facilitated by molecular chaperones in cooperation with their co-chaperones. The R2TP protein complex has emerged as a co-chaperone of Hsp90 that plays an important role in macromolecular assembly. The R2TP complex is composed of a heterodimer of RPAP3:P1H1DI that is in turn complexed to members of the ATPase associated with diverse cellular activities (AAA +), RUVBL1 and RUVBL2 (R1 and R2) families. What makes the R2TP co-chaperone complex particularly important is that it is involved in a wide variety of cellular processes including gene expression, translation, co-translational complex assembly, and posttranslational protein complex formation. The functional versatility of the R2TP co-chaperone complex makes it central to cellular development; hence, it is implicated in various human diseases. In addition, their roles in the development of infectious disease agents has become of interest. In the current review, we discuss the roles of these proteins as co-chaperones regulating Hsp90 and its partnership with Hsp70. Furthermore, we highlight the structure–function features of the individual proteins within the R2TP complex and describe their roles in various cellular processes.

## Introduction

Macromolecular complexes are crucial for driving essential processes in the cell. Because of the crowded nature of the cell, the formation of macromolecular complexes requires significant navigation to avoid the formation of non-productive associations. Molecular chaperones in general, and the Hsp90:R2TP protein complex in particular, play important roles in facilitating correct macromolecular complex assembly. The R2TP complex is composed of a heterodimer of RNA polymerase II-associated protein 3 (RPAP3):PIH1 domain-containing protein 1 (P1H1DI) that is in turn complexed to members of the ATPase associated with diverse cellular activities (AAA +), RUVBL1 and RUVBL2 (R1 and R2) (Rivera-Calzada et al. 2017; Seraphim et al. 2019). As a cellular protein complex assembly tool, the R2TP system is functionally versatile as it facilitates the formation of both protein–protein and protein-RNA complexes (Matias et al. 2006; Dauden et al. 2021**)**. Its clients span across molecules implicated in gene expression such as nuclear RNA polymerases, small nucleolar RNPs (snoRNPs), required for the formation of ribosomes, and mechanistic target of rapamycin (mTOR) complexes 1 and 2, which are implicated in translation (Boulon et al. 2012). One of the key clients of the R2TP complex is telomerase ribonucleoprotein (RNP), which plays an important role in cell proliferation (Boulon et al. 2012). The RTP complex is also implicated in processes such as chromatin remodeling, transcription, telomerase complex assembly, phosphatidylinositol-3 kinase-related protein kinase (PIKK) signaling, RNA polymerase II (RNAP II) assembly, mitotic spindle assembly, and apoptosis (Ikura et al. 2000; Shen et al. 2000; Izumi et al. 2012; Kakihara and Saeki 2014). As a co-chaperone of Hsp90, the functional versatility of the R2TP complex has earned it notable research attention (Boulon et al. 2010; 2012; von Morgen et al. 2015; Seraphim et al. 2019). Due to this functional versatility, it is not a surprise that the R2TP is essential for various cellular events (Horejsi et al. 2010; Maurizy et al. 2021).

## The AAA + protein family

**Fig. 1:**
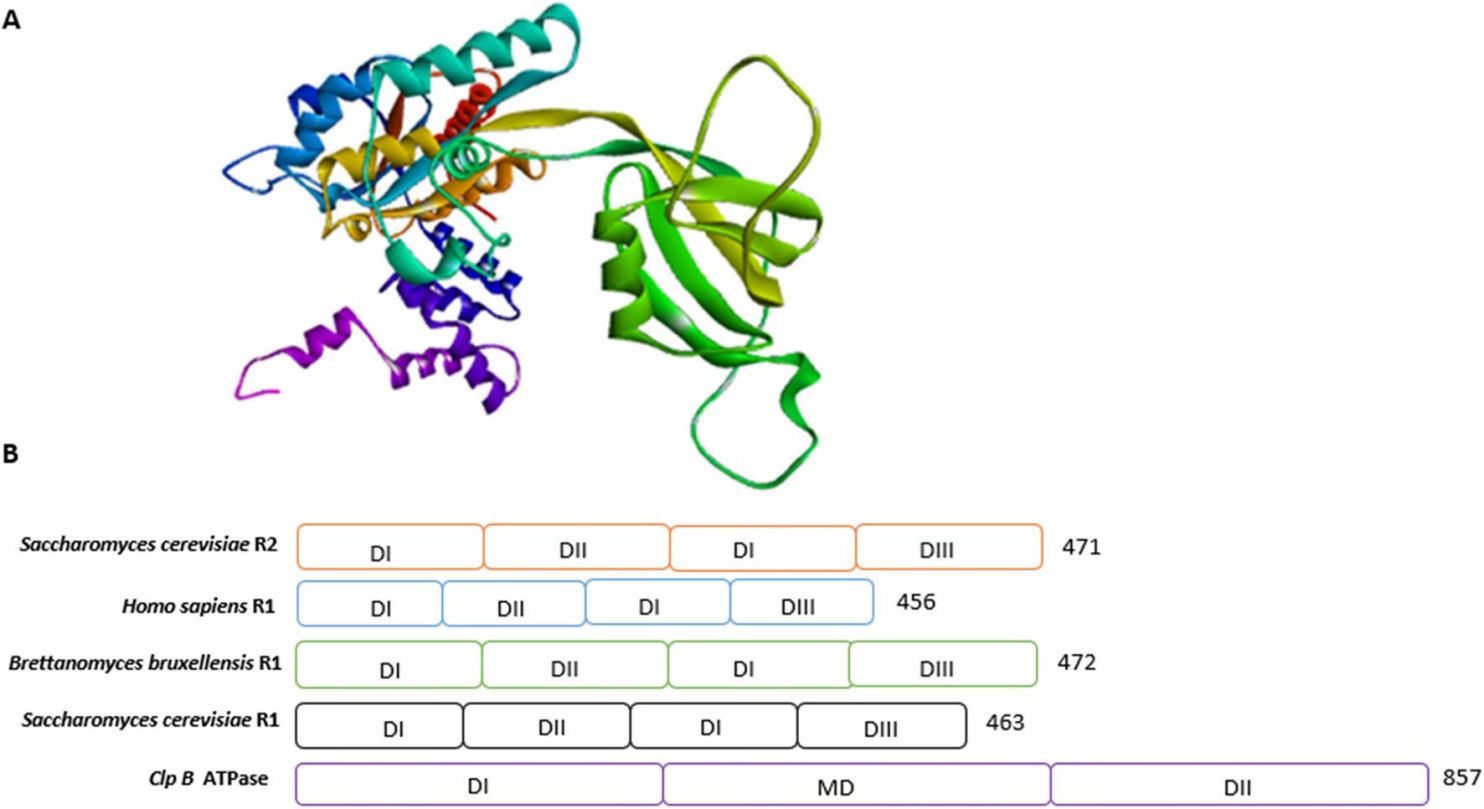
Structural organization of ATPases belonging to the ASCE sub-group of AAA + proteins. Three-dimensional model of PfRUVBL1 an ATPase protein from the parasite *Plasmodium falciparum* (**A**). The predicted functional motifs including Walker A (orange region), Walker B (light blue region), sensor 1 (blue region), and sensor 2 (dark blue region) are shown. The structures were drawn using the Blast Omega tool and rendered on the discovery studio tool (II). Schematic representation of AAA + proteins domains (**B**). Clp ATPase family of proteins possesses two ATPase domains (DI and DII), while ASCE group members have three ATPase domains (DI, DII, and DIII)

AAA + proteins are molecular chaperones defined by a highly conserved phosphate-loop nucleoside triphosphate (P-loop NTPase) (Erdmann et al. 1991; Gates and Martins 2020). The NTPase domain is constituted by an ATPase module of about 200–250 amino acids, which is characterized by an αβα core. This structure is further subdivided into a P-loop (Walker A) and a Walker B motif (Fig. 1). These domains occur as two helices arranged in a hairpin-like structure and are oriented in a left-handed orientation (Seraphim and Houry 2020). Members of the AAA + super-family belong to two groups: the kinase group (KG) and the additional strand catalytic E (ASEC) group (Seraphim and Houry 2020).

**Fig. 2:**
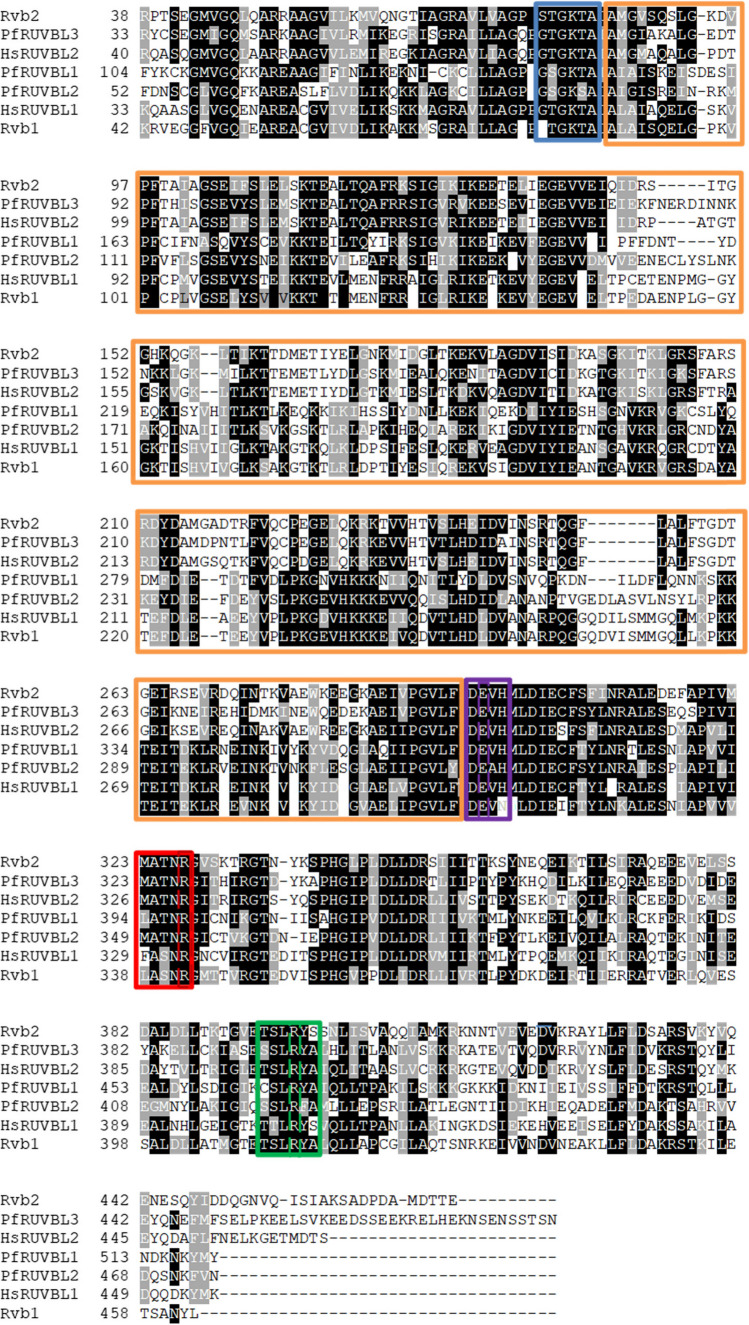
Sequence alignment of ASCE group AAA + protein of human R1 and R2 with their counterparts from yeast (Rvb1 and Rvb2) and *Plasmodium falciparum* (PfRUVBL1, PfRUVBL2, and PfRUVBL3). Functional segments such as AAA + domain, Walker A motif (blue), Walker B motif [purple) (with its glutamic acid residue, (broken purple line)], Arginine finger (broken lines), sensor 1 (red), and sensor 2 (green) are shown. Sequences were obtained from PlasmoDB and aligned using Cluster Omega and shaded using box shading tool

AAA + members belonging to the ASCE subgroup are conserved across all species from bacteria to humans (Snider et al. 2008; Seraphim and Houry 2020). The ASCE subgroup, like all P-loop NTPases, possesses a core αβα nucleotide-binding domain that exhibits two key functional features: nucleotide-binding (ATP) and hydrolysis (ATP → ADP + PO_4_^3−^) and are generally referred to as Walker A and Walker B, respectively (Ogura et al. 2004). All ASCE group members also possesses a catalytic glutamate (E) residue located within the Walker B motif (Fig. 2), while those that fall under the kinase-GTPase (KG) group exhibit Walker A and Walker B motifs adjacent to each other (Seraphim and Houry 2020). The Walker A motif is located within the P-loop, that is, in turn positioned between the β1 strand and the α-helix (Fig. 1). The Walker A motif is responsible for both ATP binding and regulates conformational changes that drive ATP hydrolysis (Ogura et al. 2004). On the other hand, the hydrophobic Walker B motif is located within the β3 strand and contributes towards ATP hydrolysis and metal ion coordination (Kanade et al. 2020). The Walker B motif is characterized by the presence of acidic residues (aspartate/glutamate) (Walker et al. 1982; Leipe et al. 2003; Kanade et al. 2020).

In addition to the Walker motifs, the ASCE members possess the following conserved segments: second region of homology (SRH), arginine finger (R-finger), sensor 1, and sensor 2 (Seraphim and Houry 2020, Figs. 1 and 2). The SRH is located between the β4 and β5 loop. On the other hand, sensor 2 is located within the third helix of the small α-helical domain and is characterized by either arginine or lysine residues located near the beginning of the α7 segment. The arginine and lysine residues regulate inter- and intra-domain communication of the protein (Neuwald et al. 1999). SRH further acts as a sensor for nucleotide binding and hydrolysis (Guenther et al. 1997). The location of the ATPase sites at the interface of two adjacent AAA + subunits offers a pathway for allosteric signal transmission and subunit coupling (Zeymer et al*.,* 2014).

## Structural organization of AAA + proteins

Typically, an AAA + protein contains a central β-sheet organized in a β5-β1-β4-β3-β2 order that is flanked by α-helicases to form an αβα sandwich (Fig. 1). With respect to domain arrangement, AAA + proteins are characterized by the presence of the non-ATPase N-terminal domain which is followed by either one or two ATPase domains: domain one (DI) and domain two (DII) (Matias et al. 2006) (Fig. 1). The ATP-binding site is located within the DI and extended to the DI-DII linker, also referred to as the middle domain (MD) (Fig. 1). One of the most notable members of the AAA + family of proteins are the Clp chaperones/proteases. The Clp family of ATPases possesses three domains: the αβα domain I (DI) and the α-helical domain III (DIII) that form the alternating AAA ring, while a central and protruding domain II (DII) represents a specific insertion located in the core AAA + domains (Aguado et al. 2015). The DI and DIII domains help to form a hexameric ring that serves as a primary feature of AAA + proteins (Wang et al. 2003). Hexamerization of the protein is crucial for ATP binding and hydrolysis (Aker et al. 2007; Ogura and Wilkinson 2001; Wang et al. 2003).

AAA + proteins play a role as transduction elements in a wide variety of functions in which energy extracted from ATP hydrolysis is used in molecular remodeling events (Snider et al. 2008). Their functions cover a diverse portfolio, including protein unfolding and degradation, peroxisome biogenesis, bacteriochlorophyll biosynthesis, DNA recombination, replication, and repair (Snider et al. 2008). In addition, compared to other P-loop ATPases, AAA + members possess an additional α-helical sub-domain which accounts for their unique structure–function features (Hanson et al. 1997). AAA + proteins mostly occur as oligomers, and function as hexameric rings (Hartman and Vale 1999). The oligomerization of AAA + proteins is stabilized largely by the binding of these proteins to nucleotides (Ogura and Wilkinson 2001). The AAA + domain is known to facilitate the oligomerization of proteins into hexameric ring-like structures (Miller and Enemark 2016). Located at the AAA + domain interface is the active site whose residues interact with residues located within a neighboring AAA + domain to drive oligomerization (Miller and Enemark 2016). Conformational changes that the hexameric protein undergoes induce ATPase activity (Kondo et al. 1997; Gates and Martins 2020).

## Proteins in the R2TP chaperone complex that are members of the AAA + superfamily

R1 and R2 are closely related AAA + proteins that share approximately 40% sequence identity (Qiu et al. 1998), and their nomenclature reflects their species of origin (Table 1). AAA + members typically form hexameric or dodecameric ring structures and are characterized by the AAA + domain, which in turn harbors the highly conserved Walker A and Walker B motifs implicated in nucleotide binding and hydrolysis, respectively (Walker et al 1982) (Fig. 1). They also possess sensors I and II and the arginine finger that are present in other AAA + proteins. Human R1 and R2 share 43% sequence identity and possess common domain organization (Dauden et al. 2021). R1 is also known as Rvb1, Pontin, Pontin52, RUVBL1, Tip49, Tip49a, NMP238, ECP54, TAP54α, TIP48, TIP49A, RVB1, and TIH1 (Gallant 2007). R2 is also known as Rvb2, Reptin, Reptin52, RUVBL2, Tip48, Tip49b, ECP51, TAP54β, CGI-46, RVB2, TIP49, TIP49β, and TIH2 (Gallant 2007).

**Table 1:** R1 and R2 members, nomenclature and their species of origin

Protein	Origin	Localization	Reference
Rvb1	*Saccharomyces cerevisiae*	Cytosol and nucleus	Zhao et al. (2005)
RUVBL1	*Homo sapiens*	Cytosol and nucleus	Qiu et al. (1998)
TIH1	*Saccharomyces cerevisiae*	Nucleus	Lim et al. (2000)
ECP54	*Caenorhabditis elegans*	Cytosol and nucleus	Salzer et al. (1999)
NMP238	*Escherichia coli*	Nuclear and cytoplasmic regions	Holzmann et al. (1998)
Tip49	*Drosophila melanogaster*	Nuclear	Bellosta et al. (2005)
Rvb2	*Saccharomyces cerevisiae*	Cytosol and nucleus	Zhao et al. (2005)
RUVBL2	*Homo sapiens*	Cytosol and nucleus	Qiu et al. (1998)
TIH2	*Saccharomyces cerevisiae*	Nucleus	Lim et al. (2000)
ECP51	*Caenorhabditis elegans*	Cytosol and nucleus	Salzer et al. (1999)

The insertion domain present within R1 and R2 resembles that of the helical1 domain present in the AAA + domain of the unfoldase chaperone, HsIU/CIpY (Bochtler et al. 2000; Wang et al. 2001). R1 and R2 assemble to form a hexameric ring, and together with their functional partners Tah1 and Pih1 form a dodecameric complex (Tsaneva et al. 1993; Matias et al. 2006). R1 and R2 are conserved across (Fig. 2) all species from humans to bacteria (Snider et al. 2008; Seraphim and Houry 2020). R1 and R2 both exhibit ATPases and helicase activities (Nano and Houry 2013). In eukaryotic cells, R1 and R2 are expressed in low abundance in relation to other components of several complexes with which they associate, and this suggests that they transiently interact with several complexes (Gallant 2007; Nano and Houry 2013).

The R2TP complex is associated with prefoldins (PFDL) and prefoldin-like constituents (Muñoz-Hernández et al. 2019). The R2TP/PFDL complex plays a crucial role in the assembly of complexes of snoRNPs, nuclear RNA polymerases, ZNHIT2, and the phosphoinositide 3-kinase (PI3K)-related protein kinase (PIKK)-containing subunits (Zhao et al. 2008; Cloutier et al. 2017). The abrogation of the expression of ZNHIT2 and R2 expression adversely impacted the protein composition of the U5 snRNP suggesting a function for these proteins in the biogenesis of ribonucleoprotein (Cloutier et al. 2017). R1 and R2 exhibit chaperone activity (Nano and Houry 2013), and their chaperone activity is important to facilitate protein assembly such as mTOR and ataxia-telangiectasia mutated (ATM; Savitsky et al. 1995). mTOR is a protein that belongs to the PIKK family and regulates translation and cell growth as influenced by cellular nutrient status (Sancak et al. 2008). On the other hand, ATM is implicated in the repair of DNA damage (Blackford and Jackson 2017). R1 and R2 have been shown to facilitate proteostasis through their capability to enhance aggresome formation and disaggregation of amyloid fibrils (Zaarur et al. 2015). Furthermore, R1 and R2 are implicated in facilitating the branch migration of holiday junctions, which occurs during homologous recombination (Putnam et al. 2001). The interaction of R1 with γ-tubulin is thought to influence mitosis (Gartner et al. 2003; Ducat et al. 2008).

Several studies have demonstrated that R1 and R2 are associated with various chromatin remodeling complexes such as the INO80 complex in *Saccharomyces cerevisiae*, *Homo* s*apiens*, and *Drosophila melanogaster* (Shen et al. 2000; Jónsson et al. 2001; Jin et al. 2005). In addition, the two proteins are thought to be involved in the formation of multi-subunit complexes, including those that are involved in processes that catalyze the deposition and removal of the histone variant, SWR-C complex (Table 1) in *S. cerevisiae* (Morrison and Shen 2009) and *Homo sapiens* (Kobor et al. 2004; Mizuguchi et al. 2004). In humans, R1 and R2 are implicated in the formation of the acetyltransferase complex (TiP60 complex), a key regulator of genome expression and stability (Kusch et al. 2004; Cai et al. 2003). The crystal structure of the AAA + subdomain of human R1 exhibits a central channel large enough to fit ssDNA but which is too small to accommodate double helix DNA, suggesting that such hexamer associates only with ssDNA (Matias et al. 2006). R1 and R2 proteins have been shown to independently form part of the chromatin remodeling complexes, INO80 (Shen et al. 2000), and SWR-C complexes (Krogan et al. 2003). Because of their roles in functional protein complex assembly, R1 and R2 are expressed in various organs such as the lungs, liver, and colon (Nano and Houry 2013). Consequently, their functional deficiency results in apoptotic cell death and various diseases including cancer (Rousseau et al. 2007; Nano and Houry 2013).

Considering the crowded nature of the cell, complex assembly essentially depends on maintaining macromolecules involved in conformations that are appropriate for assembly and these molecules must be prevented from entering non-productive associations until they are presented to the correct complex. As such, R1 and R2 combine chaperone functions while also serving as modules for protein complex assembly. The chaperone function of the R1 and R2 is regulated by an insertion domain that is located between Walker A and Walker B (Fig. 2) (von Morgen et al. 2015). It is interesting to note that RUVBL proteins are conserved in various species, including those causing infectious diseases. The main agent of malaria, *P. falciparum*, expresses members of the R2TP family (Seraphim et al. 2019). It must be noted however that *P. falciparum* is distinct in that it expresses three RUVBL proteins (Seraphim et al. 2019). In addition, *P. falciparum* lacks RPAP3/Tah1 homologue (Seraphim et al. 2019). These observations highlight the unique scope of these proteins in some organisms which suggest them for selective targeting in drug design efforts.

## Heat shock proteins

Heat shock proteins (Hsp) are conserved proteins that play an important role as molecular chaperones. In this regard, their main role is to facilitate folding of other proteins. They are so-called due to their initial discovery in response to the heat stress (Ritossa, 1962). Hsps are generally classified according to their molecular sizes in kDa. Major Hsp classes include small heat shock proteins (sHsps), Hsp40/J proteins, Hsp60, Hsp70, Hsp90, and Hsp100 (reviewed in Edkins and Boshoff 2021). The R2TP complex primarily serves as a co-chaperone of Hsp90. However, experimental evidence suggesting the possible regulation of Hsp70 by the R2TP complex is imaging. For this reason, the current review is confined to the role of the R2TP complex in the regulation of these two major Hsp members.

## R2TP as a mediator of Hsp90 function

Hsp90 client proteins are found in both prokaryotes and eukaryotes but not archaea (Zhao et al. 2005). Over 500 putative Hsp90 interactors have been reported in yeast, and include transcription factors, steroid hormone receptors, and protein kinases (Zhao et al. 2005). Hsp90 is primarily involved in cell development and cell signaling (Miyata et al. 2013). It is required for the stabilization, activation, and assembly of a diverse range of proteins and complexes involved in cellular processes (Pratt et al. 2010). The pathways that Hsp90 drives are important for cell survival, and they include regulation of transcription, cell cycle progression, centrosome duplication, telomere maintenance, siRNA- mediated gene silencing, apoptosis, mitotic signal transduction, innate immunity, and targeted protein degradation (Taipale et al. 2010). Hsp90’s capability to chaperone this very broad protein clientele is provided by the function of its various co-chaperones (Prodromou et al. 1999; Seraphim et al. 2019).

**Fig. 3:**
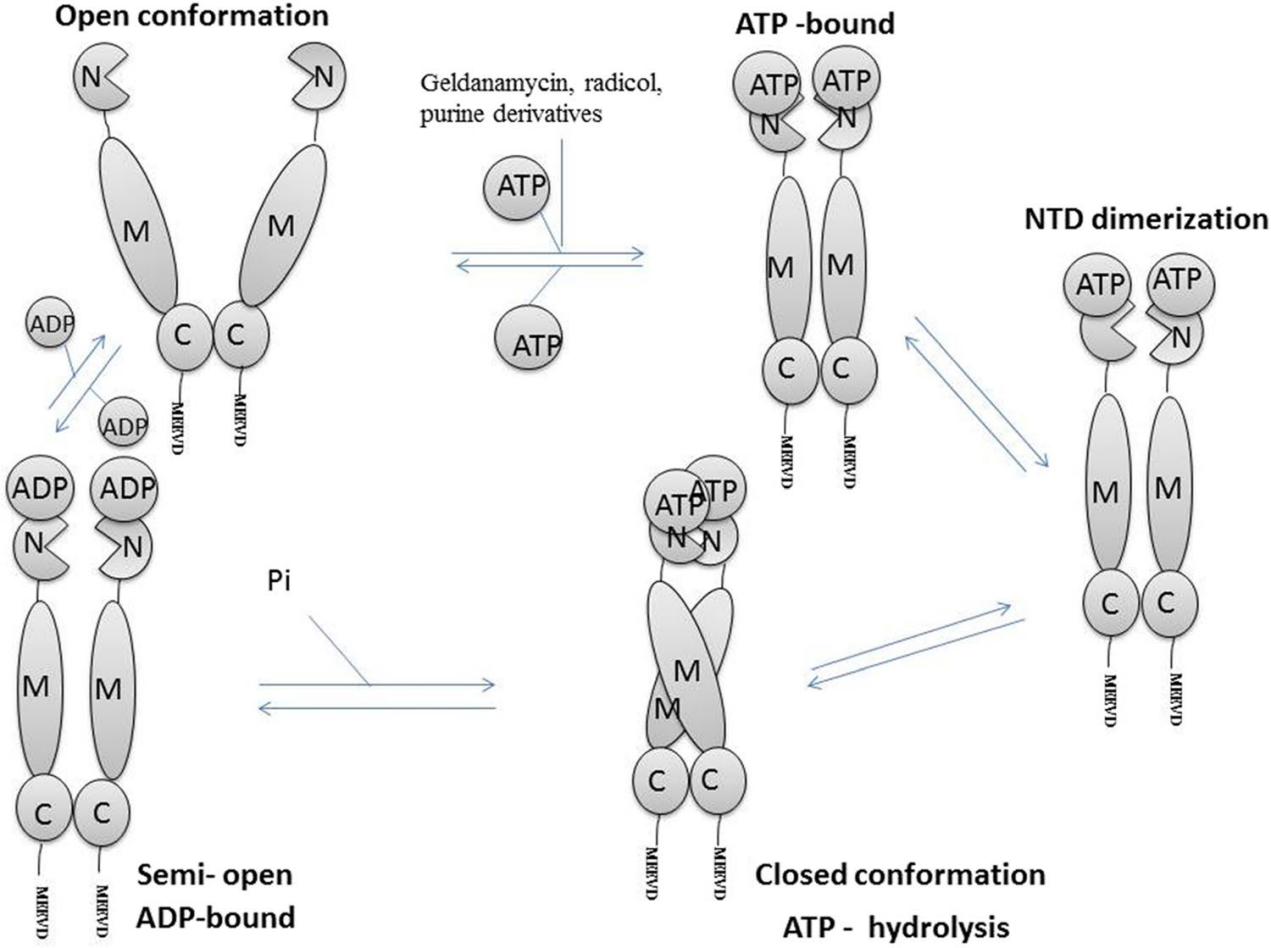
The ATPase cycle of Hsp90. Hsp90 homodimer initially adopts an open V-shape conformation. The binding of ATP to the N-terminal ATPase domain induces conformational changes culminating in the closure of the V-shaped conformation. This induces dimerization of the N-terminal domains of each protomer followed by the closure of Hsp90 and recruitment of the M-domain for ATP hydrolysis. The dimers dissociate into a semi-open intermediates state in the presence of ADP, and the release of ADP dissociates the N-termini to allow repetition of the ATP cycle. Figure was adapted from Prodromou et al. (1999) and Lackie et al. (2017)

Hsp90 is a homodimer with each subunit consisting of three domains, namely, the nucleotide-binding domain (NTD), middle domain (MD), and the C-terminal domain (CTD) (Fig. 3), and it can bind and hydrolyze ATP via its NTD (Wortmann et al. 2017). The Hsp90 ATP-dependent functional cycle involves two distinct stages. The first stage is characterized by Hsp90 forming a homodimer thus transitioning from an open V-like conformation to a compact closed one, in which the two N-terminal domains interact as directed by conformational changes associated with ATP hydrolysis (Prodromou et al. 1999; Fig. 3). ATP hydrolysis is notably low amongst the Hsp90s class, with approximately 1 ATP/min for yeast homolog (Panaretou et al. 1998). The stabilization of the open conformation of the Hsp90 is thought to be promoted by one of its crucial co-chaperones, the Hsp70-Hsp90 organizing protein (Hop) (Blatch and Lässle 1999). This leaves the second C-terminal MEEVD motif available for the binding of another chaperone allowing Hsp90 to form two distinct intermediates with TPR containing co-chaperones (Röhl et al. 2013). This invariably amplifies the functional versatility of Hsp90.

**Fig. 4:**
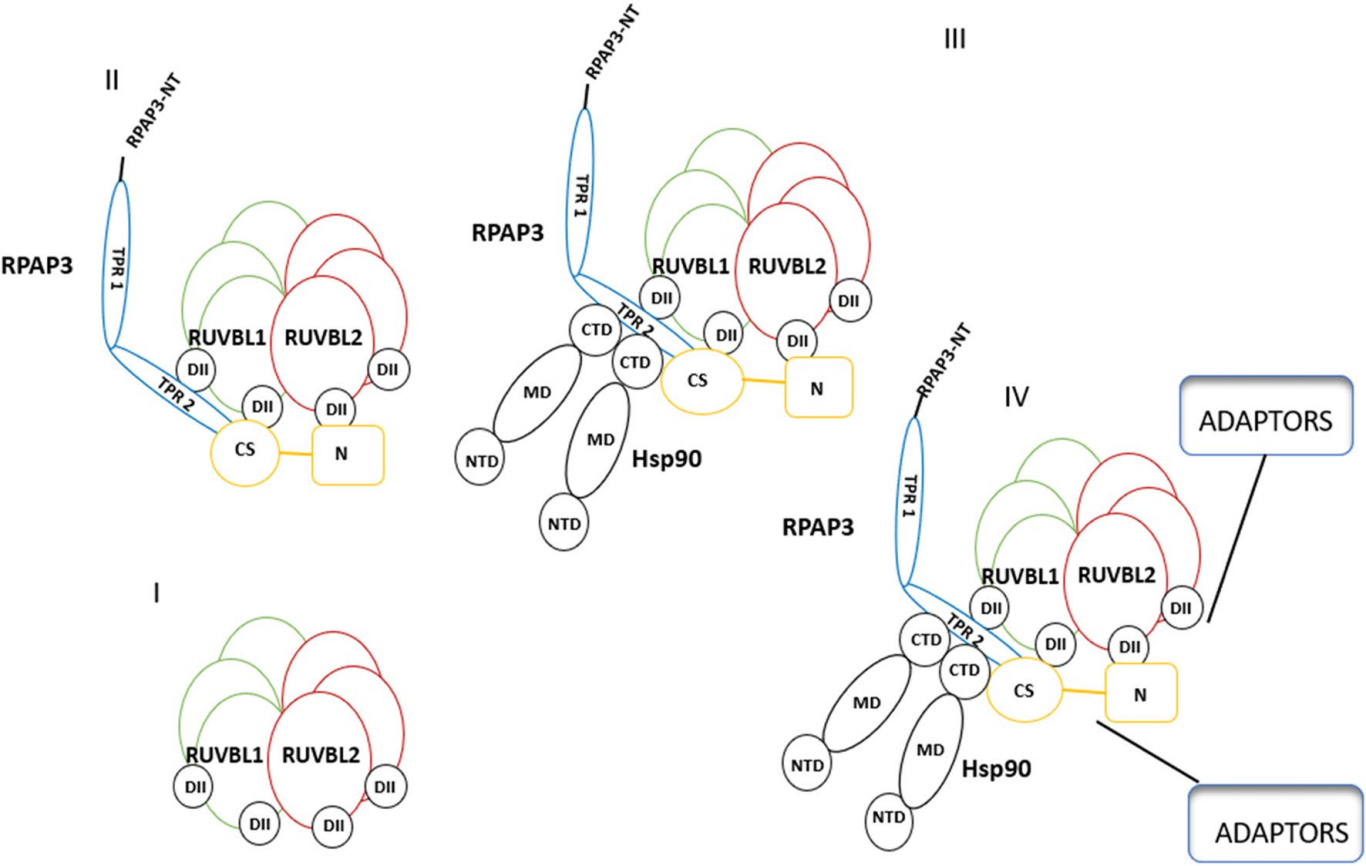
Components forming the R2TP/Hsp90 complex. Firstly, RUVBL1 and RUBL2 associate to form a hexamer of 3 subunits of each protein (Qiu et al. 1998). Tah1 and Pih1 serve as lids enclosing the AAA + hexamer (Tian et al. 2017). Pih1 acts as a scaffold connecting the R1 and R2 to Tah1, subsequently coupling the R2TP complex to Hsp90 (Pal et al. 2014). Lastly, the R2TP/Hsp90 complex client loading is facilitated by adaptors. Figure was adapted from Rivera-Calzada et al. (2017)

Protein interacting with Hsp90 (Pih1) and tetratricopeptide repeat-containing protein (Tah1) serve as lids of the AAA + hexamers of R1 and R2, respectively (Tian et al. 2017), resulting in a dodecamer R2TP complex (Fig. 4). Pih1 protein acts as a central scaffold in the R2TP complex connecting the R1/R2 proteins to Tah1 and subsequently coupling the R2TP complex to Hsp90 (Pal et al. 2014). It has been demonstrated that Tah1 and Pih1 are Hsp90 interactors (Rivera-Calzada et al. 2017), confirming their role as components of the R2TP complex (Fig. 4).

Yeast Pih1 interacts with small nucleolar ribonucleoprotein complex, snoRNP, and ribosome biogenesis-related proteins such as Rrp43, a component of the exosome, and Nop58 a component of box C/D SnoRNP (small nucleolar ribonucleoprotein complex box C motifs (RUGAUGA) and box D (CUGA) (Lakshminarasimhan et al. 2016). Nop58 is an essential nuclear protein, and Cwc24 is a zinc finger protein related to pre-U3 snoRNP splicing (Eckert et al. 2010; Aramayo et al. 2018). The human Pih1, also known as PIHIDI, interacts with box C/D snoRNP factor Nop1/fibrillarin, Nop58, Nop56, Tel2, the protein required for PIKKs stability, and WDR92/Monad which is a prefoldin-like protein containing WD40 repeats (Lakomek et al. 2015). The Monad/WDR92 is a subunit of the prefoldin complex and WD40 repeat domain (WDR) protein that is implicated in apoptosis (Saeki et al. 2006). WDR domains are β-propeller domains that serve as protein interaction scaffolds in multiprotein complexes (Schapira et al. 2017). The central cavity of the WDR domain is structurally unique for different species and hence is deemed a druggable candidate (Schapira et al. 2017). The overexpression of either Monad/WDR92 or RPAP3 in HEK293 cells enhances apoptosis and caspase-3-activation induced by tumor necrosis factor-α (TNF-α) and cycloheximide (CHX) (Itsuki et al. 2008). RPAP3, also known as hSpagh, is the human homologue of yeast Tah1 (Morgan et al. 2015). While yeast Tah1 is 111 amino acid residues long and has two TPR motifs, RPAP3 is 665 amino acid residues long and has 6 predicted TPR motifs (Taipale et al. 2010). Depletion of RPAP3 significantly reduced the induction of apoptosis, suggesting that Monad/WDR92 and RPAP3 could be modulators of the apoptotic pathways (Saeki et al. 2006). In addition, overexpression of RPAP3 promotes UV-induced cell death, while knockdown of RPAP3 decreases cell death. In contrast, the knockdown of R2 enhances cell death upon UV treatment (Inoue et al. 2010). Furthermore, depletion of PIHIDI promotes apoptosis and caspase-3 activation induced by doxorubicin in 20S osteosarcoma human cells (Inoue et al. 2010).

Yeast Pih1 is an unstable protein that is degradation prone but is stabilized by its binding to Hsp90 and Tah1 (Jiménez et al. 2012). Tah1 is composed of an N terminal domain of about 90 amino acid residues containing two tetratricopeptide repeat (TPR) motifs which are known to mediate the interaction of Hsp90 with its cofactors (Taipale et al. 2010), and the C terminal segment of about 21 amino acid residues (Pal et al. 2014).

RNA polymerases I and III synthesize non-coding RNAs that act as regulatory factors which greatly affect the growth state of the cell (White 2005). RNA polymerase II (RNAPII) synthesizes capped non-coding RNAs as well as all mRNAs (Boulon et al. 2010). This enzyme is crucial for the regulation of gene expression (Fuda et al. 2009). RNAPII is composed of 12 subunits including Rpb1 and Rpb3 which are important for the assembly of RNAPII (Boulon et al. 2010). Tah1 has been shown to directly interact with Rpb1 (the largest subunit of RNAPII) and thus possibly recruit the R2TP complex and prefoldin-like proteins together to facilitate RNAPII complex assembly (Boulon et al. 2010) (Table 2). Tah1 possesses two TPR motifs, one of which is known to recognize the C-terminal MEEVD motif of Hsp90; the C-terminus of Tah1 is the only requirement for binding to Pih1 (Zhao et al. 2008). This suggests that the TPR-containing proteins, Tah1/RPAP3, interact with proteins such as Hsp90 through the C-terminal MEEVD motif of the latter.

**Table 2:** R2TP-mediated functions of Hsp90

R2TP component	Linker	Functions	Reference
Rvb1/Rvb2	TEL2	mTORC1 stabilization	Izumi et al. (2012); Kakihara and Saeki (2014)
		SMG complex stabilization	Pal et al. (2014); Huen et al. (2010)
		ATM and DNA-PK protein complex stabilization	Cloutier et al. (2017)
		ATR/ATRIP stabilization	Machado-Pinilla et al. (2012)
	ECD	P53 stabilization	Dutta et al. (1993)
	UBR5	Transduction of DNA damage signaling	Boulon et al. (2010)
	Direct	SWR-C complex	Lakshminarasimhan et al. (2016)
	Direct	INO80 complex	Lakshminarasimhan et al. (2016)
	Direct	Tip60 complex	McKeegan et al. (2009)
	Direct	Fanconi anemia core complex	Lakshminarasimhan et al. (2016)
Pih1/PIHIDI	RPB1	RNAPII complex assembly	Boulon et al. (2010)
	TEL2	PIKKs	Horejsí et al. (2010); Takai et al. (2010)
	Rsa1/Nufip	SnoRNPs assembly	Mckeegan et al. (2009); Kakihara and Saeki (2014)
Tah1/RPAP3	Direct	SnoRNPs assembly (telomerase, ribosomes, spliceosomes)	Zhao et al. (2005)

Definition of terms in the Table 2: *UBR5*, E3 ubiquitin-protein ligase which is a nucleus cellular component; *snoRNAs*, small nucleolar ribonucleoprotein complex; SWR-C/SWR1 and INO80 are multi-subunit complexes that catalyze the admission and deletion of histone variant; *TEL2*, telomere length regulation protein; *SMG 1*, suppressor with morphological effect on genitalia 1; *ATM*, ataxia telangiectasia mutated; *ATR*, ATM and Rad3 related, *DNA-PK*, DNA-dependent-protein kinase; *RPB1*, Pol II subunit

The R2TP complex is involved in diverse cellular activities, and clients loading into the R2TP complex may either be a direct interaction with R2TP components or indirect via a linker protein (Table 2) (Kakihara and Saeki 2014). Several confirmed functions that Hsp90 undertakes through its cooperation with the R2TP complex (Table 2) have been reported (Kakihara and Saeki 2014).

## The role of R2TP in the development of cancer

**Fig. 5:**
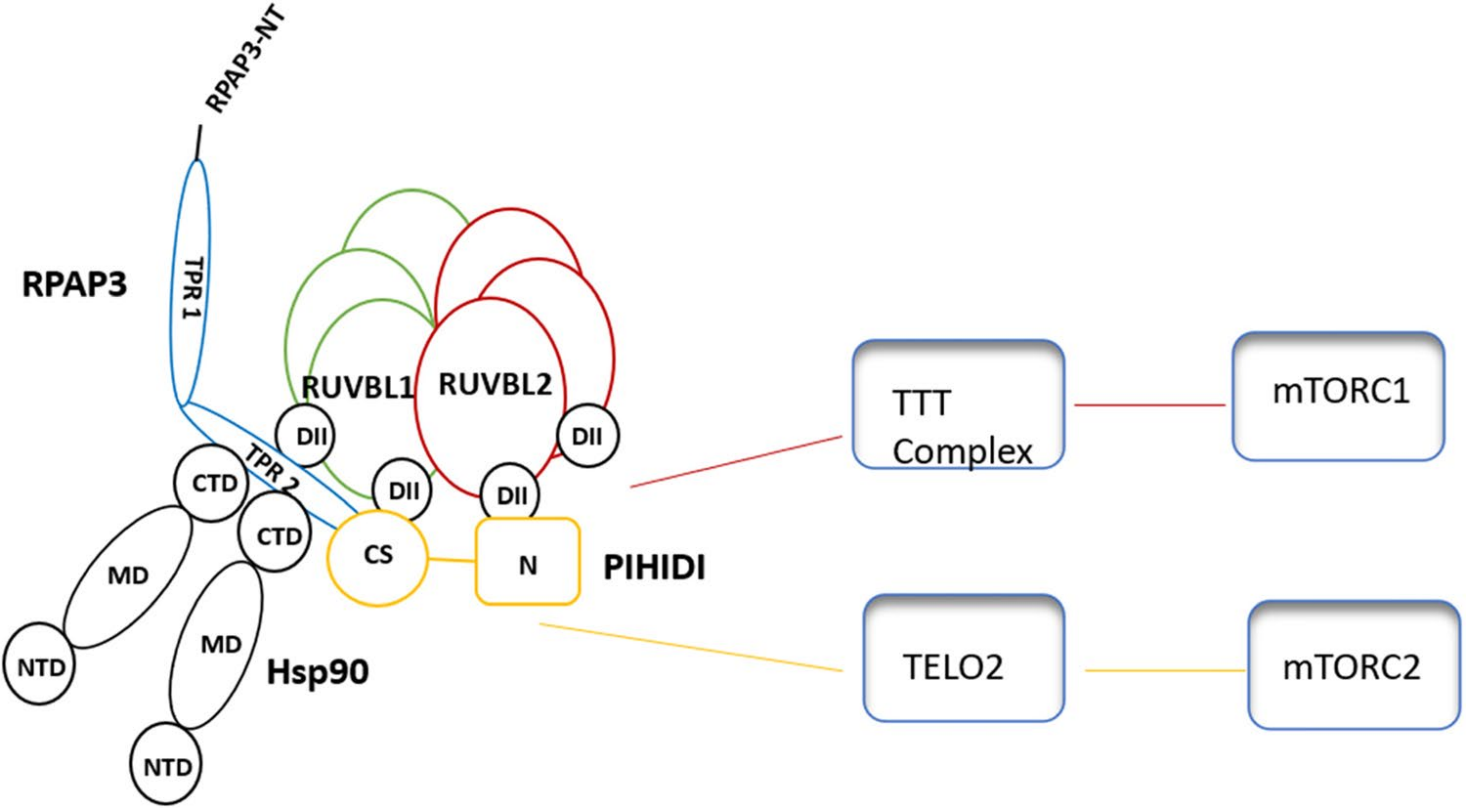
Association of the R2TP/Hsp90 complex with the mTOR complex. Direct association of R1 and R2 with mTORC1 via the TTT complex had been previously reported (Kim et al. 2013). Furthermore, the association of PIHIDI with mTORC2 had been reported (Takai et al. 2010)

The R2TP complex is important in the regulation of phosphatidylinositol-3 kinase-like kinases (PIKKs) (Kakihara and Saeki 2014). The PIKKs group consists of six members including (mTOR, SMG1, ATM, ATR, TRRAP (transformation/transcription domain-associated protein), and DNA-PKcs) (Kakihara and Saeki 2014). mTOR signaling is responsible for cell growth and proliferation, cell survival, metabolism, and protein synthesis (Tan et al. 2019). mTOR consists of two distinct complexes referred to as mTOR complex1 (mTORC1) and mTOR complex2 (mTORC2) that are both implicated in multiple diseases associated with cancer development and metabolic alterations (Tan et al. 2019) (Fig. 5). Driscoll et al. (2016) reported that mTORC2 signaling drives the development and progression of pancreatic cancer.

Both R1 and R2 reportedly occur in complex with TTT complex (TELO2-TTI1-TTI2) forming (RUVBL1-RUVBL2-TELO2-TTI1-TTI2) complex (Kim et al. 2013). They are further implicated in mTORC1 dimerization and lysosomal localization (Kim et al. 2013). The stability of the mTOR protein family is regulated by the R2TP/prefoldin complex through a linker, TELO2. Kakihara and Saeki (2014) suggested that high levels of the R2TP complex might stabilize overexpressed mTOR proteins and contribute to the malignancy of cancer.

The most virulent malignancy of mature B-lymphocytes is diffuse large B-cell lymphoma (DLBCL) which is commonly found in non-Hodgkin’s lymphoma of adults with heterogenous genetic disorders (Lone et al. 2022). This disorder is perpetuated by B-cells of the germinal center (GC) and contributes to cancer development attributes such as high cell growth rate and genomic instability (Hanahan and Weinberg 2011). An increase in cell proliferation predisposes normal cells of the B-germinal center (GCB) to malignant cancer (Monti et al. 2012). Deregulation of tumor suppressor pathways including p53 and pRb is responsible for rapid proliferation and transformation (Giacinti and Giordano 2006). In addition, the function of Rb and p53 is activated by the R2TP complex, suggesting that any alterations in any of the members of the complex can drive cells to malignancy (Lone et al. 2022).

R2TP complex functions as a regulator and suppressor of paramyxoviruses RNA synthesis (Katoh et al., 2019). Paramyxoviruses encode six or seven structural proteins and contain control regions at both genome terminals (Li and Pattnaik 1999). The viral genome has been found to associate with ribonucleoprotein (RNP) complexes which are R2TP complex clients (Park and Baines, 2006). Oncolytic viruses are considered possible anticancer therapeutic agents. One of the prospective oncolytic agents is paramyxovirus (Cattaneo 2010). It has been shown that the R2TP complex interacted with the paramyxovirus polymerase L protein and that silencing of the R2TP complex led to uncontrolled upregulation of mumps virus (MuV) gene transcription but without replication of host genome (Katoh et al., 2019). In addition, R2TP played a crucial role not only in MuV replication but also in modulated host innate immune responses (Katoh et al., 2019).

## Association of Hsp70 with the R2TP complex

Hsp70 is a molecular chaperone that is involved in several processes including protein folding, protein unfolding, assembly and disassembly of protein units, protein translocation, signal transduction, and DNA replication (Mayer and Bukau 2005; Shonhai 2021). Hsp70 function is further regulated by various post-transitional events such as cellular stress (heat stress, toxic and oxidative stress, heavy metals, and nutrient deficiency) (Rohland et al. 2022). The chaperone associates with proteins from initial folding stages to degradation, making members of the Hsp70 family central mediators of cellular proteostasis (Chakafana and Shonhai 2021). Mutations in genes encoding components of the Hsp70 system are linked to several human diseases including Parkinson’s disease, diabetes mellitus, colorectal cancer, and cardiomyopathy (Nikita et al., 2016).

Hsp70 (DnaK in prokaryotes) binds to denatured, misfolded, or aggregated proteins that display exposed hydrophobic amino acids (Mayer and Bukau 2005; Shonhai 2021). Structurally, Hsp70 possesses an N-terminal (ATPase domain) and a C-terminal (substrate-binding domain) joined by a linker. Another key function of Hsp70 is to act as a holdase chaperone, involving either the suppression of protein aggregation or the maintenance of misfolded proteins in extended form (Shonhai et al. 2008; Shonhai 2021).

**Fig. 6:**
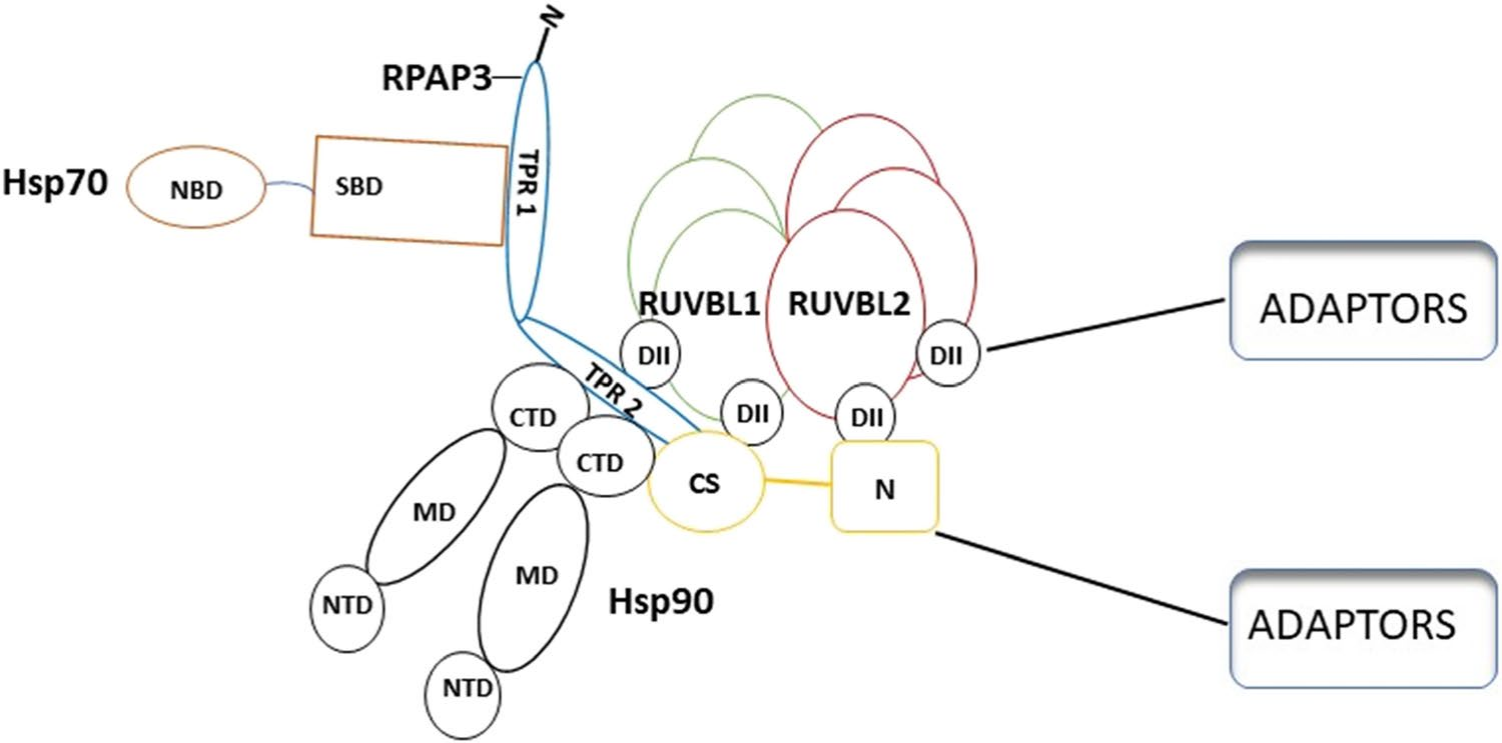
Proposed association of the R2TP complex with Hsp90 and Hsp70. Note that the TPR domains of RPAP3 make direct contact with C-terminal EEVD residues of Hsp90 and Hsp70, respectively. Definition of terms: NBD, nucleotide-binding domain; SBD, substrate-binding domain; DII, domain two; NTD, nucleotide binding domain; MD, middle domain; CTD, C-terminal domain; N, N terminus. Figure was adapted from Henri et al. (2018)

Although Hsp70 and Hsp90 function independently, they cooperate to fold specific clients such as transcription factors, kinases, and nuclear receptors (Alvira et al*.,* 2014). Hsp90 function is regulated by more than 20 co-chaperones (Pearl and Prodromou 2006). The co-chaperones bind Hsp90 at various stages of its functional cycle. STI1/Hop (Blatch and Lässle 1999) is one of its upstream co-chaperones whose function is to clamp Hsp90, thus maintaining its open conformation to allow for substrate transfer from Hsp70 (Lott et al. 2020). Downstream co-chaperones include p23 which maintains the closed conformation of Hsp90 thus allowing clients to find their full fold (Ali et al. 2006). R2TP proteins also play an important role in regulating the ATPase activity of Hsp90, thus promoting client exchange between Hsp70 and Hsp90. In addition to this, the R2TP brings Hsp90 in contact with its assembled clients (Fig. 6; Henri et al. 2018).

Hop harbors three TPR domains which enable it to bind both Hsp70 and Hsp90 and facilitate the transfer of clients from Hsp70 to Hsp90 (Blatch and Lässle 1999). Cytosol localized Hsp70 and Hsp90 possess a C-terminally located EEVD motif that interacts with Hop via its tetratricopeptide repeat (TPR) domains, TPR1 and TPR2A motifs, respectively (Scheufler et al. 2000; Assimon et al. 2015). TPR motif consists of pair of anti-parallel α-helical subdomains of about 35 amino acids (Lancaster et al. 2013). Hop possesses 9 tetratricopeptide motifs forming three TPR domains named TPR1, TPR2A, and TPR2B, and binds Hsp70 in the TPR1 and TPR2B leaving TPR2A domain preferentially binding Hsp90.

Spaghetti (Spag) is a co-chaperone found in *Drosophila melanogaster* which is a homolog of RPAP3 that possess three TPR domains named TPR1, TPR2, and TPR3 (Rodriguez and Llorca 2020). Spag binds to both Hsp90 and Hsp70 (Benbahouche Nel et al. 2014). In addition to Spag binding both Hsp90 and Hsp70, it had been shown to stimulate Hsp70 activity, further emphasizing the role of Hsp70 in the R2TP complex (Benbahouche Nel et al. 2014). RPAP3 contains two TPR domains which are proposed to bind not only Hsp90 but also Hsp70 (Henri et al. 2018). It has been suggested that Hsp70 binds to RPAP3 with medium affinity, while the RPAP3-Hsp90 complex is more favored (Henri et al. 2018). However, this remains to be experimentally demonstrated. Nonetheless, the possible interaction of RPAP3 with both Hsp70 and Hsp90 suggests that this protein acts as the bridge for client exchange between the two chaperones (Henri et al. 2018).

The association of TPR domain-containing co-chaperones with both Hsp90 and Hsp70 is well established. FKBP8, an immunophilin belonging to the FK506 family of binding proteins possesses one TPR domain and binds more tightly to Hsp90 than to Hsp70 (Blundell et al. 2017). A recent study on the regulation of prion formation demonstrated the interaction between Hsp70 and Tah1 (RPAP3 homology) as vital for regulating prion propagation and the deletion of *Tah1* was shown to improve the production of prions (Puri et al. 2021). Prion formation relies on the activity of chaperones to propagate stably in vivo (Romanova and Chermoff 2009). Hsp70 and its functional partner, Hsp104, facilitate the fragmentation of growing amyloid fibrils, thereby exposing more fibril-growing ends and generating more infectious prion seeds (Masison et al. 2009).

## Concluding remarks

The roles of R2TP proteins as regulators of proteostasis are becoming increasingly well appreciated. Their importance in protein complex assembly is crucial for diverse cellular functions including protein unfolding, protein degradation, DNA replication, peroxisome biogenesis, DNA recombination, DNA replication, and DNA repair. These central functions suggest a role for these proteins in the development of various human diseases. Indeed, their fundamental role in facilitating the development of virulence in bacteria and parasites is emerging. This further highlights the need to understand their roles in various pathways across species toward possible selective inhibition. Future research must focus on elucidating the scope of the various components constituting the R2TP complex in various species and in time and space during gene expression, translation, and macromolecular complex assembly.
